# Synthesis and Catalytic Properties of New Polymeric Monometallic Composites Based on Copolymers of Polypropylene Glycol Maleate Phthalate with Acrylic Acid

**DOI:** 10.3390/polym13244369

**Published:** 2021-12-14

**Authors:** M. Zh. Burkeev, S. R. Shibayeva, T. O. Khamitova, Jiri Plocek, Y. M. Tazhbayev, S. Zh. Davrenbekov, M. T. Nurmaganbetova, A. T. Kazhmuratova, T. S. Zhumagalieva, A. T. Kezdikbayeva

**Affiliations:** 1 Chemical Faculty, NJSC “Karaganda University Named after Academician E.A. Buketov”, Karaganda City 100000, Kazakhstan; m_burkeev@mail.ru (M.Z.B.); tazhbaev@mail.ru (Y.M.T.); sdavrenbekov@mail.ru (S.Z.D.); ritunur@mail.ru (M.T.N.); kazhmuratova@mail.ru (A.T.K.); zhumagalieva79@mail.ru (T.S.Z.); akezdikbaeva@mail.ru (A.T.K.); 2 The Department of Soil Science and Agrochemisrty, Agronomic Faculty, Kazakh Agro Technical University Named after Saken Seifullin, Nur-Sultan 010000, Kazakhstan; khamitova.t@inbox.ru; 3 Institute of Inorganic Chemistry of the Czech Academy of Sciences, Husinec-Rez 1001, 25068 Rez, Czech Republic; plocek@iic.cas.cz

**Keywords:** copolymers of polypropylene glycol maleate phthalate with acrylic acid, metal-polymer composite, electrocatalytic hydrogenation, pyridine

## Abstract

Metal-polymer composites based on copolymers of polypropylene glycol maleate phthalate with acrylic acid and metallic nickel and silver were synthesized for the first time. The objects obtained were characterized by infrared (IR) and Raman spectroscopies, thermogravimetry, a scanning electron microscope with energy dispersive spectroscopy, and atomic emission spectrometry. The catalytic activity of new metal-polymer composites that exhibited a rather high efficiency in the reactions of electrocatalytic hydrogenation of pyridine was studied. It is shown that nanoparticles of metals are evenly distributed in the volume of the polymer matrix; more than 80% of nanoparticles are in the range from 25 to 40 nm and have spherical and rhombic shapes. The reusability of the obtained composites is shown.

## 1. Introduction

In recent years, great interest has been raised by polymer–metal-immobilized systems due to their high functionality. Polymer–metal-immobilized systems combine properties such as low density, strength, and elasticity with high catalytic, magnetic properties, as well as electrical conductivity [1].

Spatially crosslinked polymeric hydrogels, which are capable of changing the size, shape, and morphology by varying external factors, including temperature, medium pH, solution ionic strength, and a mixture of aqueous–organic solvents have been used as a matrix for the metal particle immobilization [2]. In this case, a hydrogel matrix serves as a “microreactor”, in which it is possible to carry out catalytic, exchange, redox, and other types of reactions [3,4,5,6].

In addition, the increased interest of researchers in polymer-stabilized metal nanoparticles is associated with the ability to control the growth kinetics, size, and stability of nanoparticles, which ultimately determines their catalytic, magnetic, electrical, optical, biomedical, and other properties. Reducing the size of catalyst particles to nanoscale makes it possible to increase the polymer’s contact surface with the reaction medium and thereby significantly increase the catalytic efficiency, and the gel matrix protects the particles from coagulation and oxidation [7].

The need to stabilize metal nanoparticles with polymers, followed by their immobilization in the hydrogel matrix, is dictated by the fact that often, nanoparticles with a large amount of excess surface energy can be instantly passivated due to adsorption processes or coagulation [8].

To date, there are two known methods of obtaining metal nanoparticles stabilized by polymeric substances [9]. The first method consists of obtaining colloidal metal particles by thermal treatment of an aqueous–organic mixture of noble metal salts and hydrophilic polymers. However, colloidal solutions obtained in this way are unstable and tend to aggregate and precipitate during storage. The second method consists of the sorption of noble metals ions, such as gold, palladium, platinum, rhodium by hydrogels and then their reduction in the volume of the hydrogel to a zero-valent state with reducing agents.

The combination of advantages of these two methods makes it possible to stabilize metal nanoparticles with hydrophilic polymers and subsequently immobilize them in a hydrogel matrix in order to prevent migration and washout of metal nanoparticles. When using pH- and thermosensitive hydrogels, which are capable of changing their volume-phase properties depending on external stimulating factors, as a hydrogel matrix, it will be possible to finely regulate the morphology and pore sizes of the spatial network that ultimately can result in the targeted control of the metal nanoparticles behavior [10].

New mono- and bimetallic metal-polymer composites (MPCs) based on copolymers of polyethylene-(propylene)-glycol maleates with acrylic acid and metallic cobalt and nickel were synthesized earlier [11]. The possibility of using copolymers as a matrix for obtaining effective catalysts for hydrogenation has been shown. The obtained MPCs contain isolated metal NPs (MNPs), 100–112 nm in diameter and mostly spherical in shape, and are uniformly distributed in the polymer matrix [12].

The purpose of this work is to synthesize new polymer-immobilized transition metal nanoparticles based on copolymers of polypropylene glycol maleate phthalate with acrylic acid (p-PGMP/AA) with Ni and Ag and evaluate their catalytic properties in the electrocatalytic hydrogenation reaction. The main product of pyridine hydrogenation is piperidine, which is widely used for the synthesis of pharmaceuticals as a solvent, catalyst, and component of lubricating oils.

## 2. Experimental Technique

Maleic anhydride and phthalic anhydride of laboratory reagent grade, manufactured by Sigma-Aldrich (Saint Louis, MO, USA), were used without additional purification; propylene glycol was distilled by vacuum distillation. The middle fraction was dried over sodium sulfate, after which it was subjected to decantation, followed by repeated fractionation. B.p. was 470.9 K; M.p. was 260.4 K. Zinc chloride (ZnCl_2_) of analytical reagent grade, manufactured by Sigma-Aldrich (Saint Louis, MO, USA), was not subjected to additional purification; acrylic acid of laboratory reagent grade from the manufacturer Sigma-Aldrich (Saint Louis, MO, USA) was dried over calcined magnesium sulfate, followed by double vacuum distillation. B.p. was 300 K/40 mmHg. Benzoyl peroxide was dissolved in a small amount of cold chloroform and precipitated with methanol. M.p. was 107 °C. Inorganic salts (AgNO_3_, Ni(NO_3_)_2_) of laboratory reagent grade and analytical reagent grade, sodium hypophosphite of analytical reagent grade, and pyridine of laboratory reagent grade, with M.p. equal to 232.5 K and B.p. equal to 388.6 K, were used. Diethyl ether of analytical reagent grade with B.p. equal to 307.6 K/760 mmHg and M.p. equal to 256.7 K was used without additional purification.

All solvents and monomers used after purification and distillation had constants that coincided with the data given in the literature [13].

**Figure 1:**
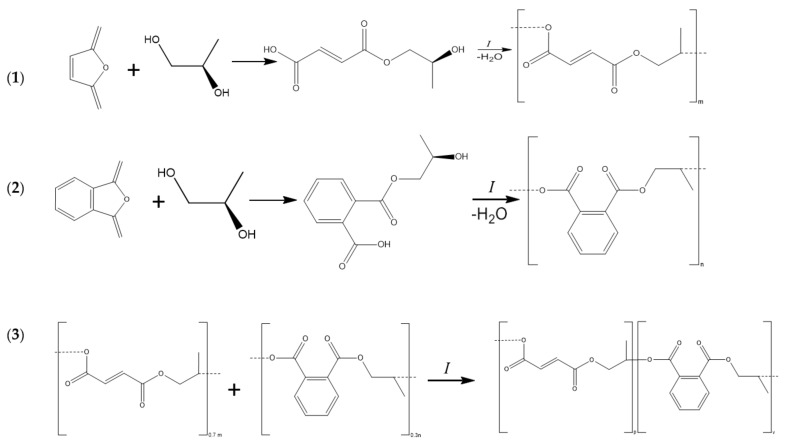
Synthesis of polypropylene glycol maleate phthalate: (**1**) the formation of an acidic ester of maleic anhydride; (**2**) the formation of acidic ester of phthalic anhydride; (**3**) obtaining polypropylene glycol maleate phthalate.

**Synthesis of polypropylene glycol maleate phthalate.** Polypropylene glycol maleate phthalate (p-PGMP) was obtained by the polycondensation reaction of propylene glycol with maleic and phthalic anhydride at a temperature of 423–433 K in a four-necked flask, which was equipped with a reflux condenser, an overhead stirrer, a thermometer, a Dean–Stark head, and a nitrogen tube. Polycondensation was carried out according to the standard procedure [14] with constant stirring in the presence of a zinc chloride catalyst in a stream of nitrogen in order to avoid gelatinization processes (Figure 1).

**Figure 2:**
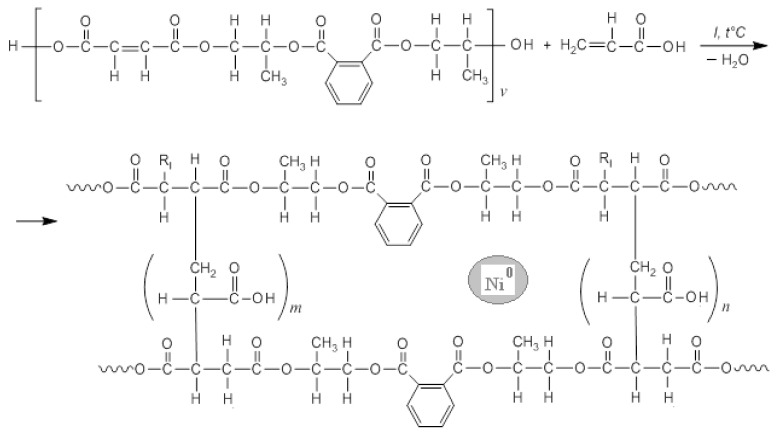
The schematic structure of polypropylene glycol maleate phthalate/AA:Ni^0^.

**Figure 3:**
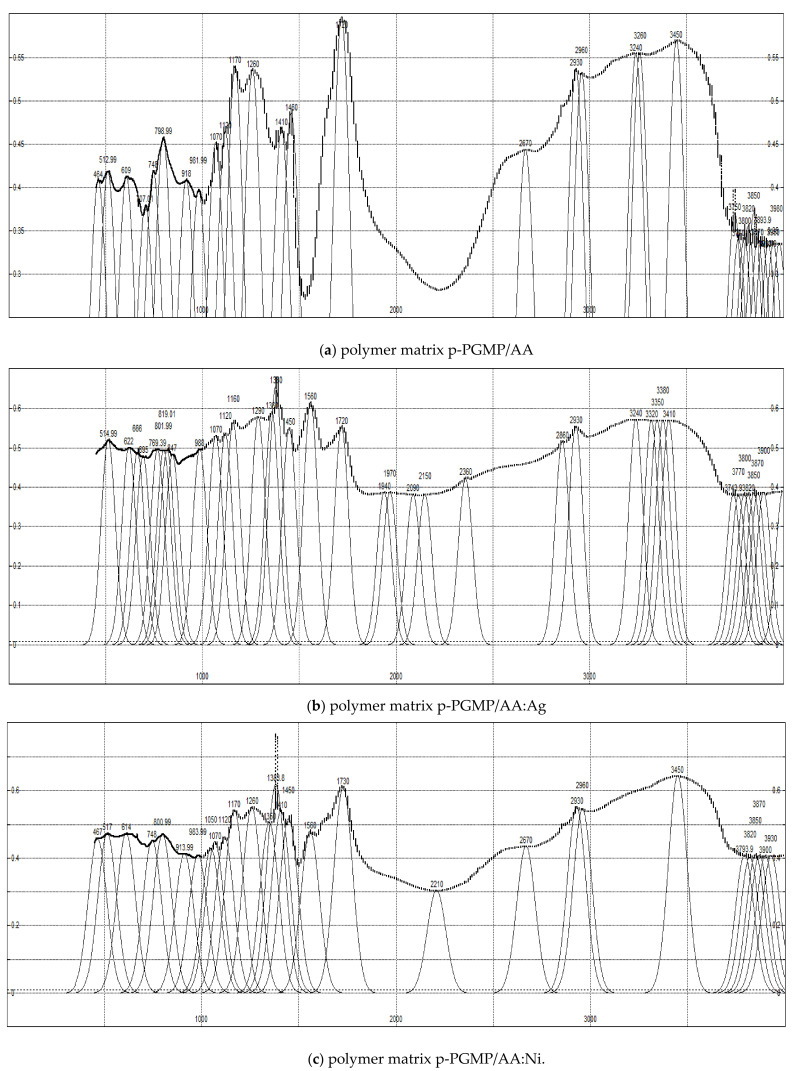
(**a**–**c**) IR spectra of samples: (**a**) polypropylene glycol maleate phthalate/AA of the composition (14.3:85.7); (**b**) polypropylene glycol maleate phthalate/AA: Ag^0^, (**c**) polypropylene glycol maleate phthalate/AA:Ni^0^.

**Synthesis of the polymer matrix**. Polypropylene glycol maleate phthalate/acrylic acid (p-PGMP/AA) was obtained by radical copolymerization, at 70 °C. Benzoyl peroxide with a concentration of 8.10^−3^ mol/L was used as an initiator (see Figure 2). The composition of the copolymers was determined by HPLC (High performance liquid chromatography), analyzing the mother liquors according to the residual principle. Composition of the polymer matrix 14.3:85.7 (weight ratio %) swelling ratio ~1200%. Copolymers were identified by means of IR data. (Figure 3a–c). Samples for IR spectroscopy were prepared by prolonged grinding of 2 ± 0.1 mg of the sample with 200 ± 0.1 mg of dry KBr; the background sample was prepared from 200 ± 0.1 mg of dry KBr. The samples were pressed under a pressure of 200 atm. The IR spectra of the obtained materials were recorded on aM FSM1201 instrument (LLC Infraspek, Saint Petersburg, Russia) with the best possible resolution of 1 cm^−1^ in the measuring mode of the relative transmission. The number of repeated scans was increased to a maximum of 100. The obtained spectra are characterized by a relatively low contrast due to the low transparency of the samples. The average transmission intensities are close for all three samples. The data were exported in the form of tables for processing in third-party software.

The spectra were inverted by subtracting the relative transmittance from unity; the positions of the absorption maxima were detected by the program, after which the amplitudes of the maxima were refined by approximating with the Gaussian contours (Figure 3a–c).

**Synthesis of metal-polymer composites—namely, polypropylene glycol maleate phthalate/AA:Ni^0^ (p-PGMP/AA:Ni^0^), polypropylene glycol maleate phthalate/AA:Ag^0^ (p-PGMP/AA:Ag^0^).** Immobilization of metal particles in p-PGMP/AA (14.3:85.7 wt.%) copolymer substrates was performed by reducing 0.5 N silver and nickel nitrate solutions. The reduction of metal ions Ni^+2^ to Ni^0^ and Ag^+^ to Ag^0^, respectively, was carried out with 0.5 N sodium hypophosphite in the presence of ammonia solution of silver chloride (5% by weight of metal) used as a catalyst. The reduction of Ni^+2^ and Ag^+^ in the polymer matrix (p-PGMP/AA) occurs in several stages. Stage 1 is the introduction of Ni^+2^, Ag^+^ ions into the polymeric matrix; stage 2 is the diffusion of reagents within the polymeric matrix; stage 3 is the reaction between nickel and silver nitrates with sodium hypophosphite, proceeding with the formation of nanosized metal particles. The reduction was carried out for 5 h at room temperature. Samples that are initially white change color, indicating immobilization of metals in the polymer volume. After that, the obtained catalyst was washed and dried to constant weight. The schematic structure of polypropylene glycol maleate phthalate/AA:Ni^0^ is shown in Figure 2.

**Figure 4:**
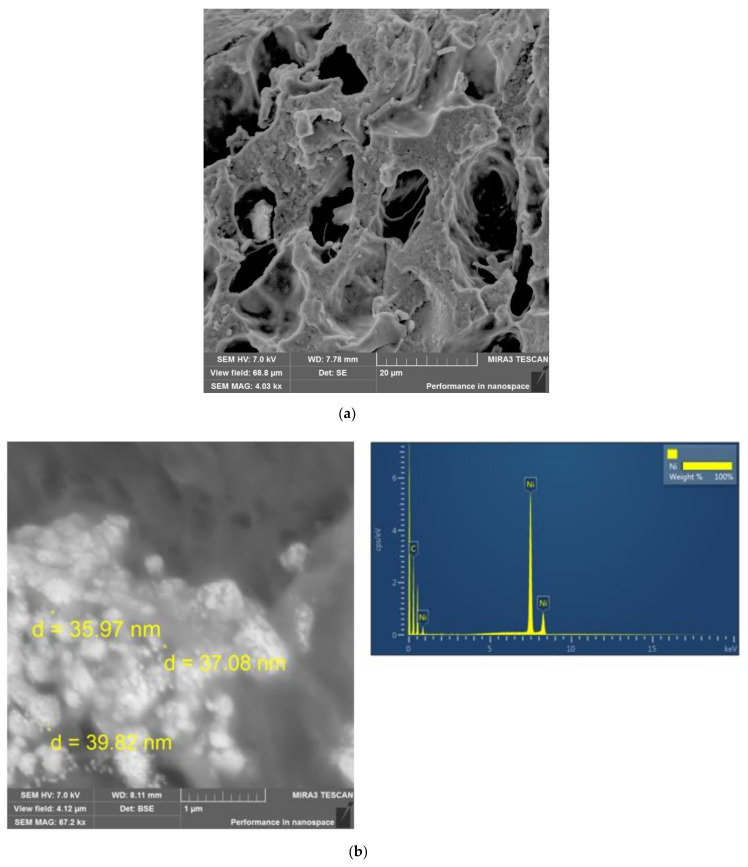

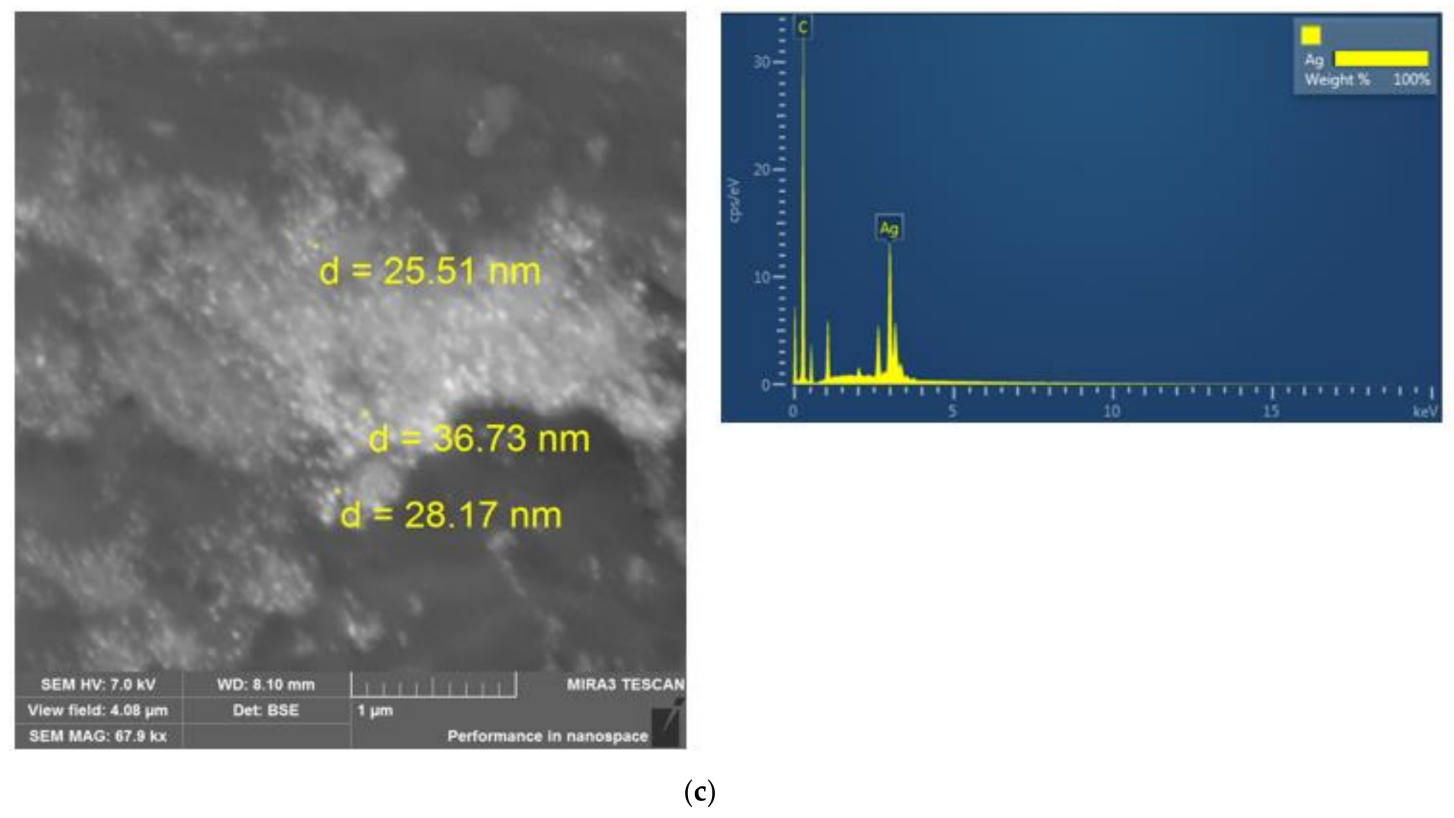
SEM image: (**a**) polypropylene glycol maleate phthalate/AA of the composition (14.3:85.7); (**b**) polypropylene glycol maleate phthalate/AA: Ni^0^, p-PGMP/AA: Ag^0^; (**c**) polypropylene glycol maleate phthalate/AA:Ag^0^.

**Physicochemical methods of metal-polymer composite (MPC) studies.** The structure, morphology, and elemental composition of the synthesized composites were studied by microscopy on a MIRA 3TESCAN Oxford Instruments SEM with a high-performance X-Act silicon drift detector (2012, Tescan Corporation, Brno, Czech Republic) for elemental analysis at an accelerating voltage of 7 kV (Figure 4).

**Figure 5:**
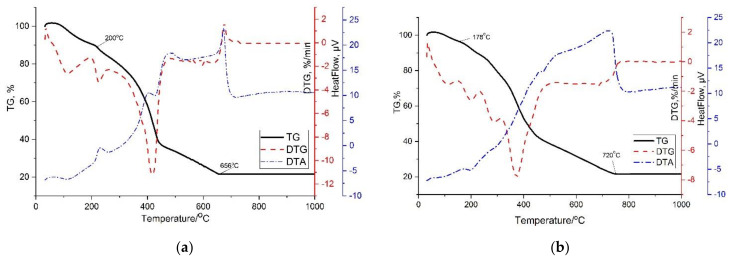
Thermogram of metal-polymer composites: (**a**) polypropylene glycol maleate phthalate/AA:Ni^0^, (**b**) polypropylene glycol maleate phthalate/AA:Ag^0^.

Prior to the study, the samples were coated with a conductive carbon layer on a Quorum Q150R ES sputtering apparatus (Quorum Technology, Lewis, UK). The amount of adsorbed metal in the composite was determined on an Agilent 4210 microwave plasma atomic emission spectrometer (MP–AES), (Agilent Technologies, Bayan Lepas Free, Malaysia), with a commercial air-cooled magnetron, operating at 2450 MHz, constant plasma power 1KW. It was determined by processing the obtained spectra, using software, and determining the mass fraction of elements by the positions and intensities of characteristic spectral lines (see Table 1). The thermal stability of the composites was investigated thermogravimetrically on a synchronous TGA/DTA/DSC analyzer (Shenzhen, China) in the temperature range of 30–1000 °C in an aluminum oxide crucible at a heating rate of 5 deg/min^−1^ in the air at a flow rate of 30 mL/min^−1^ by decomposing a 20 mg sample (Figure 5).

**Electrocatalytic hydrogenation.** The hydrogenation of pyridine was carried out in a diaphragm electrocatalytic thermostatic cell separated into anode and cathode parts by an MK-40 membrane diaphragm [15]. A platinum grid served as an anode and a copper plate (conductor of the first kind) with a surface area of 0.048 dm^2^, which tightly fitted to the bottom of the electrolyzer and served as a substrate for the catalyst, as a cathode.

Hydrogenation of pyridine was carried out at different values of current and different temperatures and included the following steps: (1) process initiation—introduction of solutions and regulation of process conditions: a 20% NaOH solution was used as an anolyte and a 5% NaOH solution was used as a catholyte; (2) saturation: the obtained metal-polymer composite was saturated with electrocatalytic hydrogen in a cathode part of the cell during 30 min until reaching the ratio between the volume of generated H_2_ and O_2_ in burets equaling 2:1; (3) hydrogenation: a sample weight of substrate (pyridine) was introduced into the cathodic part of the cell and the volume of evolved hydrogen and oxygen was recorded every 2 min, hydrogenation was performed until hydrogen absorption was stopped. The analysis of the hydrogenation reaction products was carried out on an Agilent 7890A chromatograph with a 5975C mass-selective detector. Products obtained on catholyte were extracted with diethyl ether in a 2:1 ratio.

Immobilization of nanosized metal particles in polymeric matrices preserves both technological parameters of heterogeneous catalysts (ease of separation from reaction products, possibility of regeneration, and repeated use) and, to a large extent, activity inherent in homogeneous systems, due to the formation of metal NPs in the polymeric matrix volume, which allows the relative mobility of active centers in the reaction medium to be maintained. Consequently, the metal-polymer composites have the properties of homogeneous and heterogeneous catalysts to some extent. In this regard, the synthesis and study of MPCs based on unsaturated polyester resins and metals of nanometer dimensions is an urgent task.

## 3. Results and Discussion

The structure of the synthesized monometallic polymer composites was confirmed by IR (Figure 3a–c) (cm^−1^).

The following bands can be distinguished (cm^−1^) in the IR spectrum of the copolymer:

- p-PGMP/AA (white powder): C=O stretching vibrations of p-PGMP appear at 1720 as a broadened band due to overlapping with C=O vibrations of polyacrylate units, while C-O-C vibrations appear at 1130, 1170, 1260. Band characteristic for esters and primary alcohols is found at 1070; vibrations of C=C are detected at 1410 (broadened by O-H) and 918, -CH_2_- is found at 1460, and cis-C=C appears at 707.

In the IR spectra of metal-containing samples, the near region of 700–450 cm^−1^ is little informative due to the non-selective low transparency for this wavelength range, but with an increase in the wavenumber, the signal-to-noise ratio makes it possible to confidently indicate the bands, (cm^−1^) as follows:

- p-PGMP/AA: Ag (dark-gray powder): C=O stretching vibrations of p-PGMP appear at 1720, overlapping with C=O vibrations of polyacrylate; C-O-C vibrations appear at 1120, 1160, 1290. The narrow band of intense absorption at 1380 cm^−1^ is most likely due to silver nanoparticles. Intense bands of the carboxylate anion appear at 1560 and 1350 cm^−1^. The -CH_2_- groups appear at 1450–1460 as a relatively wide band. It seems likely that the cations were represented by ammonium and sodium, which were included in the composition during the metallization of the polymer.
- p-PGMP/AA: Ni (light gray with a green tint): C=O stretching vibrations of p-PGMP, overlapping with C=O vibrations of polyacrylate, appear at 1730; C-O-C vibrations appear at 1120, 1170, and 1260. A narrow band with intense absorption at 1388 cm^−1^ is possibly due to nickel nanoparticles. Intense bands of the carboxylate anion appear at 1560 and ~1660 cm^−1^. The intensity of these bands is lower than in the silver composite because the medium is less alkaline (ammonia solution is not used) when synthesizing a nickel-containing sample.

The IR absorption spectra of p-PGMP/AA:Ni^0^, (b) p-PGMP/AA:Ag^0^ samples are very close in the positions of absorption maxima and are characterized by low contrast associated with nonselective absorption of radiation by metal particles. Compared with the spectrum of the initial polymer, significant shifts in absorption frequencies are not observed in the spectra.

The composition, structure of the synthesized samples, and dimensions of MNPs are shown in Table 1. Images were obtained using a secondary electron detector (SE detector) at an accelerating voltage (HV) of 7 kV. The appearance of pores and the noticeable predominance of a layered porous structure are seen in the images (Figure 4). The pore sizes were found to be 0.9–1.1 µm in these samples, which was typical for spatially crosslinked polymers. (All images are shown in the size of 1 microns).

Considering the electron microscopy data, it is revealed that the size of the MNP depends on the pore size of the original polymer matrix. In our previous studies, the pore size of the initial polymer matrices, poly-(ethylene)-propylene glycol maleate:AA, polypropylene glycol maleate:AA of 0.24–0.38 μm corresponds to a denser carrier structure; the particle size in these composites was 80 ± 10 nm. In the present work, polypropylene glycol maleate phthalate/AA:Ni^0^ was obtained on the basis of polypropylene glycol maleate phthalate:AA of composition 14.3:85.7 wt% (with larger pores of 0.9–1.1 µm), the particle size was 35 ± 5 nm for nickel, and the silver particle size was 25 ± 10 nm (Table 1, Figure 4).

BSE detectors were used for image acquisition. BSE shows material (compositional) contrast. The SEM images (Figure 4) show the morphology of metals in the form of spherical particles of Ag and Ni particles of rhombic shape uniformly distributed in the gel matrix. Particles with sizes from 20 to 60 nm are the main part of nanoparticles (about 80%) of the total mass. A smaller portion is accounted for by larger formations, 25–40 nm in size. Aggregates with sizes larger than 200 nm (about 10%) are formed as a result of the adhesion of small particles that are marked on the polymer surface. The metal content of Ag, Ni in p-PGMP/AA:Ag^0^, p-PGMP/AA:Ni^0^ nanocomposites is ~23, 21 wt.% of the total mass of the composite, respectively.

The results of energy dispersive spectrometry show a relatively uniform distribution of Ni^0^, Ag^0^ along the cross section of the polymer. The average number of metal particles per 1 μm is ~2000 ± 200 units of Ni particles and ~1900 ± 150 units of Ag particles (Figure 4).

Figure 5 shows thermograms with a constant heating rate of 10 deg min in the temperature range of ~30–1000 in an air atmosphere. According to the data of thermogravimetric analysis of the p-PGMP/AA–Ag^0^, p-PGMP/AA–Ni^0^ composites, the TGA curves for the composites are of the same type. The DTG curve shows an endothermic peak at 30–110 °C corresponding to the elimination of bound water, and the second peak is observed for the destruction of the p-PGMP/AA copolymer at 200–656 °C. Additionally, an intense exothermic effect is observed at a temperature of ~420 °C due to the decomposition of the compound with the removal of carboxyl groups (DTA curve). This fact is evinced by a decrease in the intensity of the bands at 1724 cm^−1^. The thermogravimetric curve of the p-PGMP/AA–Ni^0^ section at ~470–480 °C is accompanied by stabilization of the mass, and it is 500–510 °C for p-PGMP/AA–Ag^0^. The total weight loss is ~80%. Visually, this transition is accompanied by the blackening of the compounds, which is probably due to the remainder of metal nanoparticles ~20%.

**Catalytic properties of p-PGMP/AA:Ni^0^, p-PGMP/AA:Ag^0^ systems in the reaction of electrocatalytic hydrogenation of pyridine.** According to the literature data, the main way to obtain saturated nitrogen-containing heterocyclic compounds is liquid-phase catalytic hydrogenation of the corresponding pyridine bases, which is carried out under harsh conditions and with the use of active catalysts. For example, hydrogenation of pyridine is carried out in autoclaves on platinum group catalysts at temperatures of 160–200 °C and hydrogen pressures up to 6Mpa [16], since pyridine deactivates the catalyst under normal conditions. In the electrocatalytic system, the hydrogenation of pyridine occurs at normal pressure and temperature with good yields of piperidine, when skeletal Ni, Cu/Ni, and Pd/Ni catalysts are used to activate the cathode.

**Figure 6:**
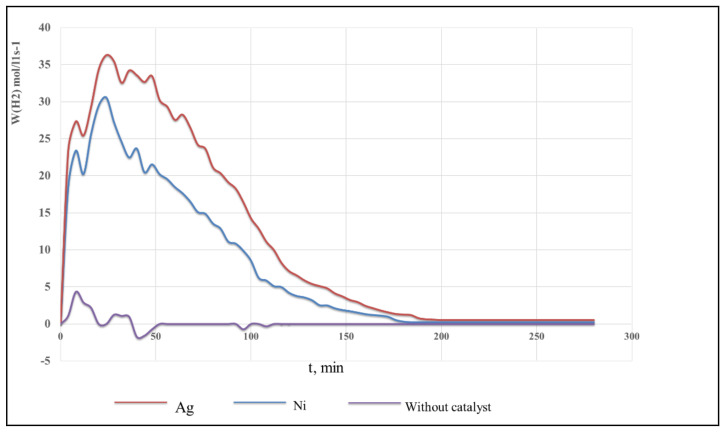
Changes in the rate of hydrogenation of pyridine during the reaction on catalysts, namely, p-PGMP/AA:Ni^0^ and p-PGMP/AA:Ag^0^.

Analyzed composites exhibit sufficiently high activity in the model reaction of electrocatalytic hydrogenation of pyridine. Thus, under comparable conditions, the reaction rate on glycol maleate phthalate/AA:Ni^0^, glycol maleate phthalate/AA:Ag^0^ is ~10 times higher (Table 2 and Figure 6).

**Figure 7:**
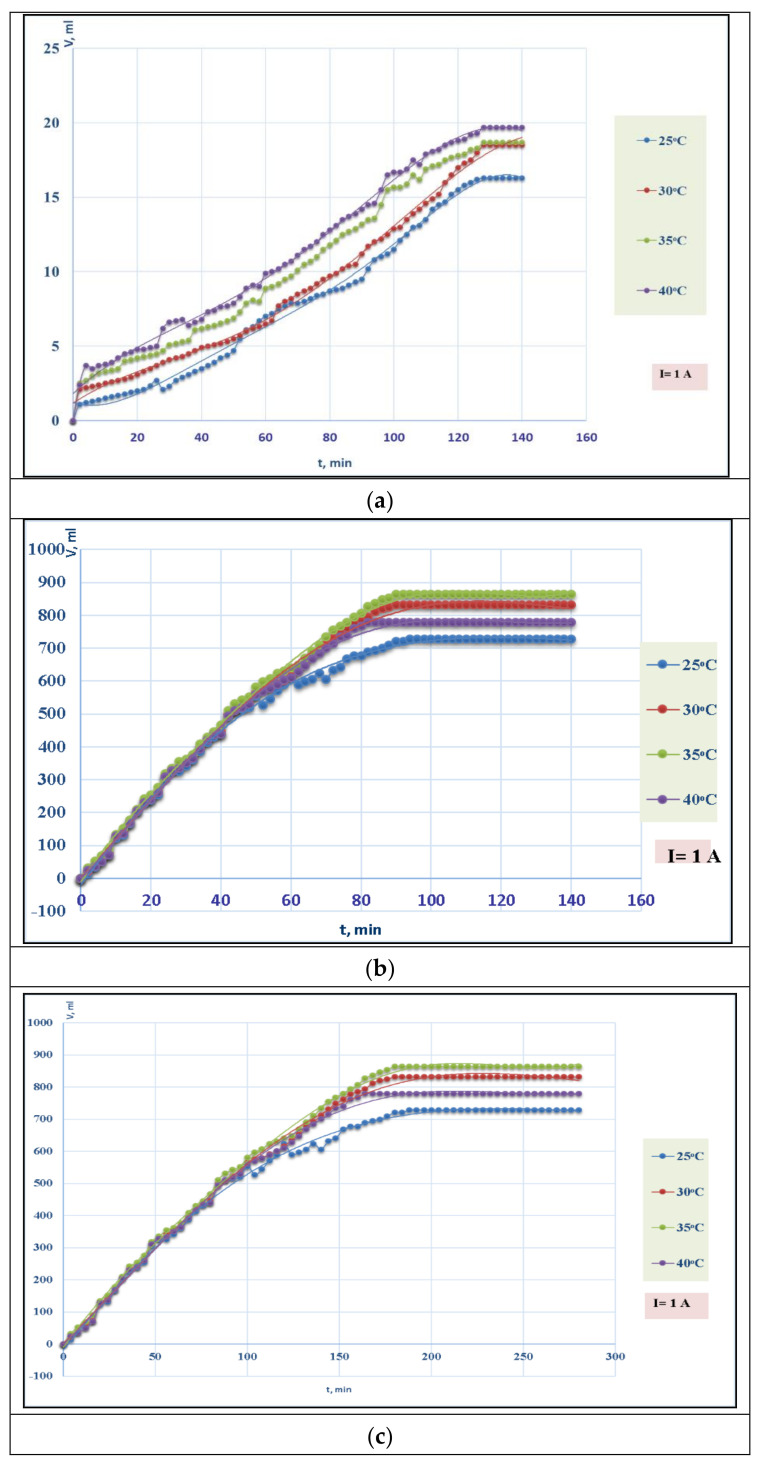
**(a**–**c**). (**a**) change in the volume of absorbed hydrogen over time without a catalyst at different temperatures; (**b**) changes in the volume of absorbed hydrogen over time in the presence of polypropylene glycol maleate phthalate/AA:Ni^0^ composite at different temperatures (°C); (**c**) changes in the volume of absorbed hydrogen over time in the presence of the polypropylene glycol maleate phthalate/AA:Ag^0^ composite at different temperatures (°C).

The curve of pyridine hydrogenation, which characterizes the process without a catalyst, is not significantly evident and lies almost parallel to the abscissa axis, while at the initial stage, the absorption of some amount of hydrogen occurs (Figure 7a). Compared with the hydrogenation process without a catalyst, the hydrogenation curve of pyridine in the presence of polypropylene glycol maleate phthalate/AA:Ni^0^ and polypropylene glycol maleate phthalate/AA:Ag^0^ shows intense hydrogen absorption. The maximum reaction rate is reached already by 20–25 min (see Figure 7), and then, it drops sharply. Within this time, in the system involving polypropylene glycol maleate phthalate/AA:Ni^0^, the pyridine is hydrogenated to 40% in the reaction products, and to 45% with polypropylene glycol maleate phthalate/AA:Ag^0^ [17]. Secondary products such as tetrahydrapyridine (4 to 7%) and dipiperidyl (5 to 7%) are found among the reaction products besides the end product piperidine.

As a continuation of the analysis, having considered the fact that polypropylene glycol maleate phthalate/AA, used as a matrix, was sensitive to temperature changes, hydrogenation of pyridine in the presence of these catalysts was carried out at various temperatures in the range of 25–40 °C (Figure 7b,c).

It should be noted from the experimental data (see Figure 7b,c) that an increase in temperature from 25 to 35 °C leads to an increase in the reaction rate; however, there is a decrease in the volume of released oxygen with further increasing temperature. This is probably connected with the fact that the polymer base begins to decompose at higher temperatures under the influence of atomic oxygen. The optimal temperature for electrocatalytic hydrogenation of pyridine on polypropylene glycol maleate phthalate/AA:Ni^0^ and polypropylene glycol maleate phthalate/AA:Ag^0^ catalysts is 30–35 °C.

**Figure 8:**
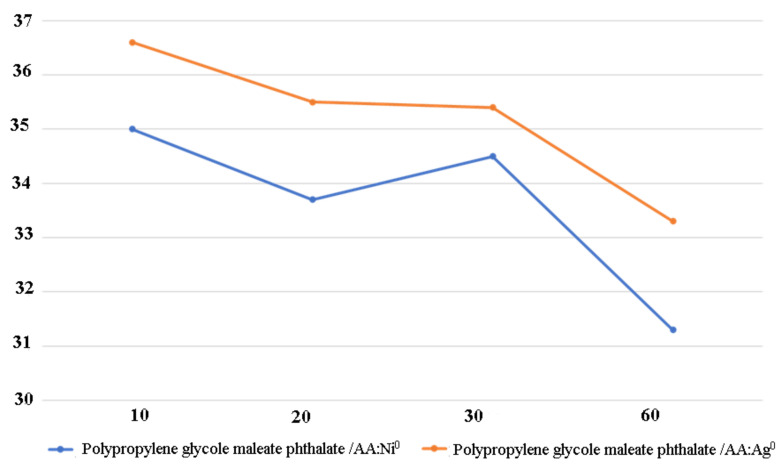
Results of reuse of polypropylene glycol maleate phthalate/AA:Ni^0^ and polypropylene glycol maleate phthalate/AA:Ag^0^.

The studied MPCs retain their catalytic activity even when repeated reaction cycles are performed on them (Figure 8, Table 3). The fact that they are in immobilized form allows them to be easily separated from the reaction medium and used repeatedly and, most importantly, to study the centers formed on the catalyst by various physical and chemical methods.

In some cases, an increase in activity is observed during repeated use, i.e., the so-called “catalyst development” characteristic of many immobilized systems occurs. (Figure 8, Table 3).

## 4. Conclusions

The pore size of the polymer matrix affects the diameter and size distribution of metal nanoparticles. The polymer matrix is a copolymer based on polypropylene glycol maleate phthalate with acrylic acid, which provides uniform distribution of metal nanoparticles and prevents the agglomeration of particles in the polymer volume. Obtained hybrid polymer-immobilized Ni, Ag nanoparticles proved to be effective catalysts for hydrogenation of unsaturated compounds. Catalytic properties of polypropylene glycol maleate phthalate/AA:Ni^0^ and polypropylene glycol maleate phthalate/AA:Ag^0^ composites depend on the conditions of their production, which obviously affect the size of the formed Ni, Ag nanoparticles. Increasing the current and temperature sufficiently affect the course of the electrocatalytic hydrogenation of pyridine. The composites studied retain catalytic activity in repeated cycles. In general, it is worth noting the necessity of such studies of the structure of metal-polymer composites, the mechanism of electrocatalytic hydrogenation with an application, and elucidation of some important aspects determining the essence and action of new polymer–metal catalysts. Further studies will focus on the dependence of the reaction rate and selectivity on the size and amount of metal NPs and on the use of nanocomposites based on other metals in hydrogenation reactions of other substrates.

## Figures and Tables

**Table 1:** Main characteristics of the synthesized monometallic polymer composites.

Metal Polymer Composite	Pore Size of Polymer Matrix, nm	Metal Content in the Composite, wt% Calculated…/pr.	Particles Size, nm	Morphology of NPs	The Average Number of Nanoparticles per 10 µm
p-PGMP/AA:Ni^0^	1000 ± 200	33/21	35±5	rhombus	2000 ± 200
p-PGMP/AA: Ag^0^	1000 ± 200	33/23	25±10	sphere	1900 ± 150

**Table 2:** Experimental data and results of pyridine hydrogenation with p-PGMP/AA:Ni^0^, p-PGMP/AA:Ag^0^ catalysts, and without catalyst.

Catalyst	T, °C	Current A	Pressure, Pa kg·m^−1^⋅s^−2^	Hydrogenation Products, %			Reaction Rate W, mol/L^1^s^−1^
				Pyridine	Piperidine	Secondary Products (Dipiperidyl)	
Without catalyst	25	1	94430	90.8	9.2	-	2.8
		1.5	94563	91.1	8.9	-	3.3
	30	1	94696	90.3	9.7	-	3.1
		1.5	94962	87.8	12.2	-	3.0
	35	1	95228	91.2	9.8	-	5.0
		1.5	95494	94.6	5.4	-	5.2
	40	1	95627	93.2	6.8	-	3.8
		1.5	95760	91.0	9.0	-	4.0
p-PGMP/AA:Ni^0^	25	1	95494	46.3	41.7	9.0	22.2
		1.5	95494	45.0	43.5	8.5	25.4
	30	1	95893	29.8	61.0	7.2	31.0
		1.5	96026	27.0	62.5	8.5	34.2
	35	1	95893	20.3	64.0	11.0	32.4
		1.5	95893	22.2	64.6	9.2	35.0
	40	1	95760	41.5	46.2	10.3	24.3
		1.5	95627	33.7	50.5	10.8	26.6
p-PGMP/AA:Ag^0^	25	1	95494	39.4	49.1	9.5	24.2
		1.5	95760	34.2	52.8	9.0	25.3
	30	1	95627	27.1	62.2	8.7	34.0
		1.5	95494	27.2	62.8	8.0	36.0
	35	1	95361	24.0	63.5	9.5	34.5
		1.5	95627	24.8	65.0	8.2	36.6
	40	1	95095	36.8	51.0	10.2	26.5
		1.5	94962	34.1	54.2	9.7	28.4

**Table 3:** Results of MPC reuse.

Reuse	Reaction Rate W, mol^−1^s^−1^		Yield of the Target Product, %
	Polypropylene Glycol Maleate Phthalate/AA:Ni^0^	Polypropylene Glycol Maleate Phthalate/AA: Ag^0^	
After 10 days	35.0	36.6	50.5
After 20 days	33.7	35.5	48.1
After 30 days	34.5	35.4	47.2
After 60 days	31.3	33.3	42.4

Experimental conditions: T = 35 °C, I = 1.5 A, p = Hg (≠const).
